# The impact of intrahepatic cholestasis on pregnancy outcomes: a retrospective cohort study

**DOI:** 10.1186/s12876-023-02652-3

**Published:** 2023-01-18

**Authors:** Yiming Chen, Huimin Zhang, Wenwen Ning, Yijie Chen, Caihe Wen

**Affiliations:** 1 Department of Prenatal Diagnosis and Screening Center, Hangzhou Women’s Hospital (Hangzhou Maternity and Child Health Care Hospital), No. 369, Kunpeng Road, Shangcheng District, Hangzhou, 310008 Zhejiang China; 2 The Fourth School of Clinical Medical, Zhejiang Chinese Medical University, Hangzhou, 310053 Zhejiang China; 3 Department of Obstetrics, Hangzhou Women’s Hospital (Hangzhou Maternity and Child Health Care Hospital), Hangzhou, 310008 Zhejiang China

**Keywords:** Intrahepatic cholestasis of pregnancy, Fetal intrauterine growth restriction, Thrombocytopenia, Hyperlipidemia, Premature rupture of membranes, Low birth weight infants, Odds ratio, Pregnancy outcomes

## Abstract

**Background:**

This study analyzed the pregnancy outcomes of patients with intrahepatic cholestasis of pregnancy (ICP) in Hangzhou, China.

**Methods:**

Cases of pregnant women monitored by antepartum testing at Hangzhou Women’s Hospital from January 2018 to December 2020 were reviewed. Subjects were classified into two groups according to whether they had ICP: 688 cases of ICP were assigned to an exposure group while 38,556 cases of non-ICP were assigned to a non-exposed group. Univariate analysis was performed on qualitative or quantitative data using the Chi-Squared test or Mann–Whitney *U* test, and the adjusted odds ratio (aOR) and 95% confidence interval (CI) of the two groups of related variables were calculated by multivariate binary logistic regression analysis.

**Results:**

The incidence rate of ICP was 1.75%. Pregnant women with hepatitis B virus were correlated with ICP. Hepatitis B carriers (aOR = 3.873), preeclampsia (PE, aOR = 3.712), thrombocytopenia (aOR = 1.992), gestational hypertension (GH, aOR = 1.627), hyperlipidemia (aOR = 1.602) and gestational diabetes mellitus (GDM, aOR = 1.265) were all risk factors for ICP. In contrast, Body Mass Index (BMI) ≥ 30 kg/m^2^ (aOR = 0.446), 25 m^2^ < maternal BMI < 29.9 kg/m^2^ (aOR = 0.699) and parity ≥ 1 (aOR = 0.722) were protective factors for ICP. Pregnant women in the ICP group had an increased risk of gestation days < 259 days (aOR = 4.574) and cesarean delivery (aOR = 1.930) after ICP, and a decreased risk of longer gestational days (aOR = 0.105), premature rupture of membranes (aOR = 0.384) and fetal macrosomia (aOR = 0.551).

**Conclusions:**

By analyzing a Chinese population with ICP, we identified that pregnant women who are hepatitis B carriers or with PE, thrombocytopenia, GH, hyperlipidemia, and GDM are at higher risk of ICP. Moreover, ICP is associated with adverse pregnancy outcomes; in particular, ICP may increase the incidence of shorter gestational days and non-vaginal delivery methods such as cesarean section but reduce the incidence of premature rupture of membranes and fetal macrosomia.

**Supplementary Information:**

The online version contains supplementary material available at 10.1186/s12876-023-02652-3.

## Background

Intrahepatic cholestasis of pregnancy (ICP) is a common pregnancy-specific liver disease that usually presents in the second trimester. ICP is clinically characterized by maternal pruritus without a rash and abnormal liver function tests, including abnormal levels of serum bile acids (≥ 10 µmol/L). The incidence of ICP is approximately 0.50–2.00% of all pregnant women and varies widely among certain ethnic groups. ICP is associated with an increased risk of adverse perinatal outcomes, including spontaneous preterm birth, the contamination of amniotic fluid with meconium, and stillbirth [1]. Because of its globally recognized efficacy and safety, ursodeoxycholic acid is currently the main choice for treating ICP [2]. It must also be mentioned that a serum level of bile acids > 40 µmol/L could increase the risk for the fetus, hence, bile acid monitoring should be performed throughout pregnancy [3].

At present, the etiology of ICP remains unknown but may be closely related to a range of factors such as maternal age, twin and multiple pregnancies, genetics, estrogen levels and immunity. In a previous study, Yue pointed out that the down-regulation of iNOS and the up-regulation of NPY may affect the blood supply between the uterus, placenta and the fetus in ICP and that this may account for acute hypoxia and adverse pregnancy outcomes [4]. A review article by Shan et al. [5] proposed that the existence of autophagy may play a role in the etiology and prevention of ICP. In addition, it has been suggested that delivery at 37 weeks of gestation may be better because fetal death due to ICP appears to mainly occur after 37 weeks [6].

The effects of ICP on pregnant women are mild, however, ICP may be complicated by fetal arrhythmias, fetal hypoxia, premature birth, and even in severe cases, death in utero. However, uncertainty remains as to the relationship between ICP and abnormal pregnancy outcomes. An 8-year case-controlled study showed that the adjusted odds ratio (aOR) for respiratory distress syndrome and neonatal morbidity was 2.56 fold higher in an ICP group than in a non-ICP group after adjustment for confounders. However, the rate of postpartum hemorrhage was twice as high in the ICP group as in the non-ICP group [7]. Another large prospective cohort study demonstrated a significantly increased risk of adverse perinatal outcomes, including stillbirth among pregnant women with severe ICP (Total Bile Acid, TSBA ≥ 40 µmol/L). Therefore, it is recommended that we strengthen the prenatal monitoring of pregnant women with severe ICP [8]. These previous studies proved that ICP is related to adverse pregnancy outcomes. However, some reports found that ICP was associated with adverse perinatal outcomes that could not be predicted by routine fetal monitoring [9, 10].

Therefore, we conducted a retrospective cohort study, including 39,244 pregnant women, of which 688 cases had been diagnosed with ICP, to analyze the impact of ICP on pregnancy outcomes in Hangzhou, China.

## Methods

### Cohort selection

A total of 39,244 pregnant women were included in this retrospective study. These patients underwent inpatient delivery in the obstetrics department of Hangzhou Women’s Hospital between January 2018 and December 2020. Specifically, there were 688 cases with ICP (the exposure group) and 38,556 cases without ICP (the non-exposure group). Each pregnant woman was routinely tested for routine blood analysis, routine urine analysis, liver function, renal function, thyroid function, bile acids, systolic blood pressure, diastolic blood pressure, and other parameters after admission. In addition, all research subjects were singletons and conceived naturally. This study was approved by the Hangzhou Women’s Hospital Medical Ethics Committee (2020-Yilunshen A No. 10–11). This research has obtained informed consent from the patients.

### Diagnosis and exclusion criteria

#### Case diagnosis

According to the requirements of the ICP Diagnosis and Treatment Guidelines (2015) [11], patients were diagnosed by the presence of pruritus and a bile acid level ≥ 10 µmol/L. In addition, the diagnosis was made according to biochemical and other clinical and auxiliary examination results of ICP. Pregnancy complications included hypertensive disorders of pregnancy (HDP), gestational diabetes mellitus (GDM), thrombocytopenia, hyperlipidemia and pregnancy-associated anemia. Various pregnancy outcomes were assessed, including premature rupture of membranes (PPROM), cesarean section, fetal intrauterine growth restriction (IUGR), fetal distress, premature birth, low birth weight, and fetal macrosomia. All pregnancy complications and pregnancy outcomes, in line with the corresponding Chinese guidelines, were obtained from clinical records, as diagnosed by hospital obstetricians [12–15].

HDP include gestational hypertension (GH) and preeclampsia (PE); these are maternal and perinatal factors that represent the leading causes of death. Preeclampsia was defined as blood pressure values of ≥ 140/90 mmHg accompanied by proteinuria, which referred to either ≥ 300 mg/24 h urinary protein or ≥ 30 mg/dL in random urine samples [16].

IUGR is the failure of a fetus to achieve its designated growth potential; this is related to fetal or maternal factors [17, 18]. Low birth weight (LBW) was defined as an infant weight < 2500 g. Fetal macrosomia was defined as an infant weight > 4000 g. Apgar scores referred to the average of the scores at 1, 5 and 10 min.

Advanced maternal age was defined as an expected gestational age ≥ 35 years of age, while young pregnant women were defined as an expected gestational age < 35 years. For the convenience of calculation, we converted the gestational weeks into gestational days in a uniform manner, and three groups [< 259 days, Normal (259–287 days) and > 287 days] were divided according to whether the delivery was premature or overdue. Body Mass Index (BMI) was divided into thin (< 18.5 kg/m^2^), normal (18.5–25 kg/m^2^), obese (25–29.9 kg/m^2^) and obesity (≥ 30 kg/m^2^) according to the World Health Organization (WHO).

### Exclusion criteria

**Fig. 1:**
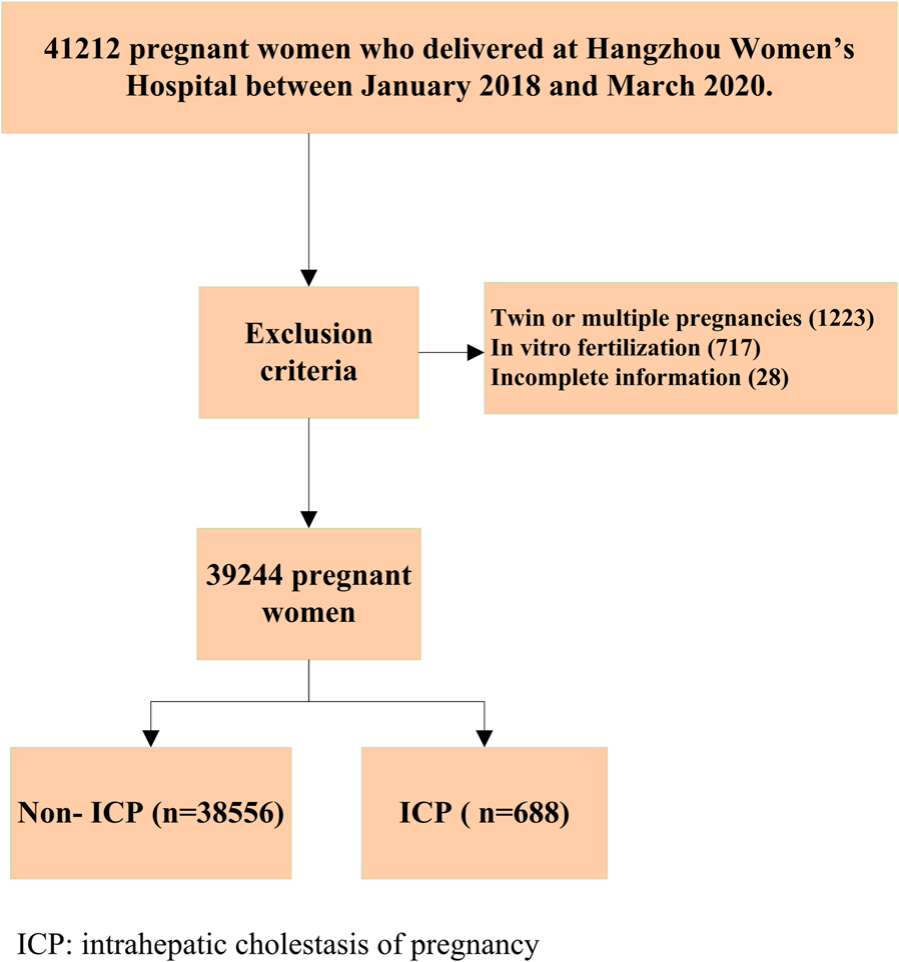
Flowchart of 39,244 pregnant women who were divided into non-ICP and ICP groups

To reduce the influence of twin pregnancy and in vitro fertilization (IVF) on the results, we excluded cases involving twin or multiple pregnancies, IVF infants, and those with an incomplete dataset, see Fig. 1.

### Statistical analysis

Statistical analysis was performed by IBM-SPSS 24.0 statistics (IBM-SPSS, Chicago, USA). Univariate analysis of qualitative or quantitative data was performed using the Chi-Squared test or Mann–Whitney *U* test, with *P* < 0.10 as the selection criteria for multivariate binary logistic regression analysis. Multivariate binary logistic regression analysis was used to screen the variable odds ratio (OR) and 95% confidence interval (CI) of each relevant influencing factor, adjusted odds ratio (aOR), after adjusting for potential confounding variables (Backward: Likelihood Ratio Test (LR): Based on all candidate variables, the independent variables that do not meet the requirements of the significant retention level are eliminated from the model at one time, and the remaining variables are entered into the model [19]). The variable input in step 1 included: before the occurrence of ICP: gravidity ≥ 1, parity ≥ 1, hypertensive disorders of pregnancy (HDP) [gestational hypertension (GH), preeclampsia (PE)], hyperlipidemia, gestational diabetes mellitus (GDM), anemia, thrombocytopenia, hepatitis B virus carriers, BMI (Categories); after the occurrence of ICP: premature rupture of membranes (PPROM), fetal growth retardation, preterm birth, gestational days (Categories), caesarean section, infant sex (female), infant length (cm), infant weight (Categories). Differences were considered statistically significant when *P* < 0.05.

## Results

### A comparison of maternal demographics

There were 688 cases of ICP among 39,244 pregnant women (the exposed group), and the incidence rate of ICP was 1.75%. The median maternal age of participants in the two groups was 29.00 years; there was no significant between the two groups (*Z* = 0.062, *P* = 0.951). Nevertheless, the maternal weight, gestational days at delivery, gravidity and parity in the ICP group were all significantly lower than those in the non-ICP group (all *P* < 0.001). In addition, the incidence of non-vaginal delivery methods such as cesarean section in the ICP group was significantly higher than that in the non-ICP group (*P* < 0.001), as shown in Table 1.

**Table 1:** Univariate analysis of maternal demographic in the ICP and non-ICP groups

Indicators	Groups		*Z*/*x*^2^	*P*
	Non-ICP (*n* = 38,556)	ICP (*n* = 688)		
Maternal age (years)	29.00 (23.00–38.00)	29.00 (23.00–38.00)	0.062	0.951
Advanced maternal age (years)			0.103	0.748
No	34,601 (89.74)	620 (90.09)		
Yes	3955 (10.26)	68 (9.91)		
Maternal weight (kg)	67.00 (53.00–87.00)	65.00 (52.00–83.00)	7.112	< 0.001*
Maternal height (cm)	160 (151.00–170.00)	160 (150.00–170.00)	2.632	0.008**
BMI (kg/m^2^)	25.97 (21.10–32.87)	25.37 (20.46–31.65)	6.019	< 0.001*
Categories (BMI)			30.356	< 0.001*
Thin (< 18.5 kg/m^2^)	56 (0.15)	3 (0.44)		
Obese (25–29.9 kg/m^2^)	20,445 (53.03)	327 (47.53)		
Obesity (≥ 30 kg/m^2^)	4126 (10.70)	48 (6.98)		
Normal (18.5–25 kg/m^2^)	13,929 (36.13)	310 (45.05)		
Gestational days	273.00 (250.00–287.00)	266.00 (234.00–280.00)	20.667	< 0.001*
Categories (Gestational days)			270.511	< 0.001*
< 259 days	1862 (4.83)	128 (18.60)		
> 287 days	427 (1.11)	1 (0.15)		
Normal (259–287 days)	36,267 (94.06)	559 (81.25)		
Systolic blood pressure (mmHg)	118.00 (98.00–138.00)	117.00 (98.00–139.00)	1.397	0.162
Diastolic blood pressure (mmHg)	73.00 (60.00–92.00)	74.00 (58.00–93.00)	2.660	0.008**
MAP (mmHg)	87.33 (74.00–106.00)	88.00 (71.83–107.00)	1.209	0.227
Gravidity			13.014	< 0.001*
0	18,957 (49.17)	386 (56.10)		
≥ 1	19,599 (50.83)	302 (43.90)		
Parity			14.187	< 0.001*
0	25,727 (66.73)	506 (73.54)		
≥ 1	12,829 (33.27)	182 (26.46)		
Mode of delivery			120.899	< 0.001^*^
Natural childbirth	26,376 (68.40)	335 (48.69)		
Caesarean section	12,180 (31.60)	353 (51.31)		

ICP, Intrahepatic cholestasis of pregnancy; BMI, Body Mass Index; MAP, maternal mean arterial pressure; *P < 0.001; **P < 0.05

### A comparison of neonatal demographics

The weight and length of newborns in the ICP group were significantly lower than those in the non-exposed group (*P* < 0.001). However, there was no statistically significant difference between the groups in terms of the mean Apgar score and gender of newborns. (*P* > 0.05), as shown in Table 2.

**Table 2:** Univariate analysis of newborns demographics in the ICP and non-ICP groups

Indicators	Groups		*Z*/*x*^2^	*P*
	Non-ICP (*n* = 38,556)	ICP (*n* = 688)		
Infant weight (g)	3300 (2400–4110)	3120 (1982–4000)	11.023	< 0.001*
Categories (infant weight)			76.384	< 0.001*
Low birth weight infants (< 2500 g)	1291 (3.35)	64 (9.30)		
Fetal macrosomia (≥ 4000 g)	1551 (4.02)	15 (2.18)		
Normal (2500–4000 g)	35,714 (92.63)	609 (88.52)		
Infant length (cm)	50 (48–51)	50 (45–50)	13.345	< 0.001*
Infant Apgar score	10 (9–10)	10 (9–10)	1.668	0.095
Infant sex			3.458	0.063
Female	20,086 (52.09)	383 (55.67)		
Male	18,470 (47.91)	305 (44.33)		

ICP, Intrahepatic cholestasis of pregnancy; *P < 0.001

### Univariate analysis of influencing factors in the two groups of pregnant women

Tables 1, 2, 3 and 4 shows that univariate analysis revealed several factors were related to ICP (*P* < 0.10), such as BMI, diastolic blood pressure, gravidity, parity, mode of delivery, gestational days, infant weight, infant length, Apgar score, HDP, hyperlipemia, preterm birth, GDM, anemia during pregnancy, thrombocytopenia, premature rupture of membranes, fetal growth retardation and hepatitis B carriers. The incidence of other factors was not significantly different between the two groups (*P* > 0.10).

**Table 3:** Clinical characteristics of the ICP and non-ICP groups

Factors	Non-ICP	ICP	*x*^2^	*P*
	*n* = 38,556	*n* = 688		
HDP			15.424	< 0.001*
GH	1317 (3.42)	31 (4.51)		
PE	602 (1.56)	34 (4.94)		
Normal blood pressure	36,637 (95.02)	623 (90.55)		
GDM			3.640	0.056
No	33,151 (85.98)	574 (83.38)		
Yes	5405 (14.02)	114 (16.62)		
Thyroid function			0.617	0.734
Hypothyroidism	3163 (8.21)	62 (9.01)		
Hyperthyroidism	70 (0.18)	1 (0.15)		
Normal thyroid function	35,323 (91.61)	625 (90.84)		
Hyperlipidaemia			8.674	0.003**
No	37,096 (96.20)	647 (94.04)		
Yes	1460 (3.80)	41 (5.96)		
Anemia			4.435	0.035
No	29,246 (75.86)	498 (72.38)		
Yes	9310 (24.14)	190 (27.62)		
Thrombocytopenia			10.072	0.002**
No	38,038 (98.66)	669 (97.24)		
Yes	518 (1.34)	19 (2.76)		
Amniotic fluid volume			1.091	0.580
Oligohydramnios	2075 (5.38)	31 (4.51)		
Polyhydramnios	209 (0.54)	4 (0.58)		
Normal amniotic fluid volume	36,272 (94.08)	653 (94.91)		
Uterine scar			0.022	0.882
No	33,607 (87.16)	601 (87.35)		
Yes	4949 (12.84)	87 (12.65)		
Placental abruption			0.377	0.539
No	38,368 (99.51)	683 (99.27)		
Yes	188 (0.49)	5 (0.73)		
Placenta previa			1.395	0.238
No	38,261 (99.23)	680 (98.83)		
Yes	295 (0.77)	8 (1.17)		
Uterine atony			0.940	0.332
No	37,106 (96.24)	667 (96.95)		
Yes	1450 (3.76)	21 (3.05)		
Pregnant women with hepatitis B carrying status			147.443	< 0.001*
No	36,979 (95.91)	595 (86.48)		
Yes	1577 (4.09)	93 (13.52)		

ICP, Intrahepatic cholestasis of pregnancy; HDP, hypertensive disorders of pregnancy; GH, gestational hypertension; PE, preeclampsia; GDM, gestational diabetes mellitus; *P < 0.001; **P < 0.05

**Table 4:** Pregnancy outcomes of pregnant women in the ICP and non-ICP groups

Pregnancy outcomes	Non-ICP	ICP	*x*^2^	*P*
	*n* = 38,556	*n* = 688		
Premature rupture of membranes			55.100	< 0.001*
No	29,746 (77.15)	613 (89.07)		
Yes	8810 (22.85)	75 (10.93)		
Fetal distress			0.097	0.755
No	34,947 (90.64)	626 (90.99)		
Yes	3609 (9.35)	62 (9.01)		
Cord entanglement			2.305	0.129
No	26,874 (69.71)	498 (72.38)		
Yes	11,682 (30.29)	190 (27.62)		
Preterm birth			263.303	< 0.001*
No	36,680 (95.13)	560 (81.40)		
Yes	1876 (4.87)	128 (18.60)		
Fetal growth retardation			6.771	0.009**
No	38,359 (99.49)	679 (98.69)		
Yes	197 (0.51)	9 (1.31)		
Postpartum hemorrhage			0.083	0.773
No	38,449 (99.72)	687 (99.85)		
Yes	107 (0.28)	1 (0.15)		
Stillbirth			0.738	0.390
No	38,388 (99.56)	687 (99.85)		
Yes	168 (0.44)	1 (0.15)		

ICP, Intrahepatic cholestasis of pregnancy; *P < 0.001;**P < 0.05

### Results of multi-factor binary logistic regression analysis

The results of multifactorial binary logistic regression analysis showed that hepatitis B carriers (aOR = 3.873), PE (aOR = 3.712), thrombocytopenia (aOR = 1.992), GH (aOR = 1.627), hyperlipidemia (aOR = 1.602) and GDM (aOR = 1.265) were risk factors for ICP. In contrast, BMI ≥ 30 kg/m^2^ (aOR = 0.446), 25 kg/m^2^ < maternal BMI < 29.9 kg/m^2^ (aOR = 0.699) and parity ≥ 1 (aOR = 0.722) were protective factors for ICP. Other factors were not associated with the risk of ICP, as shown in Table 5.

**Table 5:** Further binary logistic analysis of maternal characteristics and pregnancy complications before the diagnosis of ICP

Variants	ICP		OR	95% CI for OR	*P*	aOR	95% CI for aOR	Adjusted *P*
	Non (*n* (%))	Yes (*n* (%))						
Gravidity								
0	18,957 (49.17)	386 (56.10)						
≥ 1	19,599 (50.83)	302 (43.90)	0.757	0.650–0.881	< 0.001*	0.857	0.697–1.055	0.145
Parity								
0	25,727 (66.73)	506 (73.54)						
≥ 1	12,829 (33.27)	182 (26.46)	0.721	0.608–0.856	< 0.001*	0.722	0.607–0.858	< 0.001*
HDP								
Normal blood pressure^#^	36,637 (95.02)	623 (90.55)			< 0.001*			< 0.001*
GH	1317 (3.42)	31 (4.51)	1.384	0.961–1.994	0.081	1.627	1.123–2.357	0.010**
PE	602 (1.56)	34 (4.94)	3.321	2.330–4.734	< 0.001*	3.712	2.580–5.341	< 0.001*
Hyperlipidemia								
No	37,096 (96.20)	647 (94.04)						
Yes	1460 (3.80)	41 (5.96)	1.610	1.169–2.217	0.004**	1.602	1.160–2.212	0.004**
GDM								
No	33,151 (85.98)	574 (83.38)						
Yes	5405 (14.02)	114 (16.62)	1.218	0.994–1.492	0.057	1.265	1.030–1.554	0.025**
Anemia								
No	29,246 (75.86)	498 (72.38)						
Yes	9310 (24.14)	190 (27.62)	1.199	1.012–1.419	0.035**	1.169	0.987–1.386	0.071
Thrombocytopenia								
No	38,038 (98.66)	669 (97.24)						
Yes	518 (1.34)	19 (2.76)	2.086	1.311–3.317	0.002**	1.992	1.248–3.179	0.004**
Hepatitis B virus carriers								
No	36,979 (95.91)	595 (86.48)						
Yes	1577 (4.09)	93 (13.52)	3.665	2.929–4.587	< 0.001*	3.873	3.089–4.857	< 0.001*
BMI (Categories)								
Thin (< 18.5 kg/m^2^)	56 (0.15)	3 (0.44)	2.407	0.749–7.732	0.140	2.211	0.681–7.177	0.187
Obese (25–29.9 kg/m^2^)	20,445 (53.03)	327 (47.53)	0.719	0.614–0.841	< 0.001*	0.699	0.596–0.819	< 0.001*
Obesity (≥ 30 kg/m^2^)	4126 (10.70)	48 (6.98)	0.523	0.385–0.710	< 0.001*	0.446	0.325–0.612	< 0.001*
Normal^#^ (18.5–24.9 kg/m^2^)	13,929 (36.13)	310 (45.05)			< 0.001*			< 0.001*
Constant			0.020		< 0.001*	0.019		< 0.001*

^a^Variable (s) entered on step 1: were Gravidity ≥ 1, Parity ≥ 1, HDP (GH, PE), Hyperlipidemia, GDM, Anemia, Thrombocytopenia, Hepatitis B virus carriers, BMI. HDP, Hypertensive disorders of pregnancy; GH, Gestational hypertension; PE, Preeclampsia; GDM, Gestational diabetes mellitus; BMI, Body Mass Index; OR, odds ratio; aOR, adjusted odds ratio; CI, confidence interval. ^#^Reference; *P < 0.001; **P < 0.05

Table 6 shows that the preponderance ratios for events such as gestational days < 259 (aOR = 4.574) and cesarean section (aOR = 1.930) were 4.574, 1.930, respectively, in patients with ICP compared to those without ICP. Pregnancies with ICP were more likely to have these events.

**Table 6:** Further binary logistic analysis of pregnancy outcome after diagnosis of ICP

Variants	ICP		OR	95% CI for OR	*P*	aOR	95% CI for aOR	adjusted *P*
	Non (*n* (%))	Yes (*n* (%))						
Premature rupture of membranes								
No	29,746 (77.15)	613 (89.07)						
Yes	8810 (22.85)	75 (10.93)	0.413	0.325–0.526	< 0.001*	0.384	0.300–0.491	< 0.001*
Fetal growth retardation								
No	38,359 (99.49)	679 (98.69)						
Yes	197 (0.51)	9 (1.31)	2.581	1.318–5.056	0.006	0.989	0.476–2.056	0.977
Gestational days (Categories)								
< 259 days	1862 (4.83)	128 (18.60)	4.460	3.660–5.434	< 0.001*	4.574	3.555–5.885	< 0.001*
> 287 days	427 (1.11)	1 (0.15)	0.152	0.021–1.083	0.060	0.105	0.015–0.753	0.025**
Normal^#^ (259–287 days)	36,267 (94.06)	559 (81.25)			< 0.001*			< 0.001*
Mode of delivery								
Natural childbirth	26,376 (68.40)	335 (48.69)						
Caesarean section	12,180 (31.60)	353 (51.31)	2.282	1.962–2.654	< 0.001*	1.930	1.652–2.255	< 0.001*
Infant sex								
Female	20,086 (52.09)	383 (55.67)						
Male	18,470 (47.91)	305 (44.33)	1.155	0.992–1.344	0.063	1.150	0.987–1.340	0.073
Infant length (cm)	50 (48–51)	50 (45–50)	0.877	0.852–0.903	< 0.001*	0.987	0.940–1.037	0.610
Infant weight (Categories)								
Low birth weight (< 2500 g)	1291 (3.35)	64 (9.30)	2.907	2.234–3.784	< 0.001*	0.904	0.650–1.256	0.546
Fetal macrosomia (≥ 4000 g)	1551 (4.02)	15 (2.18)	0.567	0.339–0.949	0.031**	0.551	0.328–0.924	0.024**
Normal^#^ (2500–4000 g)	35,714 (92.63)	609 (88.52)			< 0.001*			0.065
Constant			0.025		0.003	0.013		< 0.001*

^a^Variable (s) entered on step 1: Premature rupture of membranes, Fetal growth retardation, Preterm birth, Gestational days (Categories), Caesarean Section, Infant Sex (male), Infant length (cm), Infant weight (Categories). OR, odds ratio; aOR, adjusted odds ratio; CI, confidence interval. ^#^Reference; *P < 0.001; **P < 0.05

Conversely, the preponderance ratios for events for pregnant women in the ICP group such as longer gestation days (aOR = 0.105), premature rupture of membranes (aOR = 0.384) and fetal macrosomia (aOR = 0.551) were 0.105, 0.384 and 0.551. Non-ICP patients were at higher risk for these events.

## Discussion

This study found that the positive rate of ICP among pregnant women in Hangzhou, China was 1.75%. We also found that pregnant women who were hepatitis B carriers had a higher risk of ICP. Moreover, thrombocytopenia, GH, hyperlipidemia, and GDM are also risk factors for ICP. Maternal characteristics such as a parity ≥ 1, 25 kg/m^2^ < BMI < 29.9 kg/m^2^ and BMI ≥ 30 kg/m^2^ may be protective factors for ICP. We found that pregnant women with ICP can avoid adverse pregnancy outcomes through non vaginal delivery methods, such as cesarean section and shorter pregnancy days; however, pregnant women with ICP had a decreased risk of premature rupture of membranes and fetal macrosomia.

Studies have shown that the incidence of ICP ranges from 0.02 to 2.40% and that there are large differences between regions and ethnic groups [20]. In this study, the positive rate of ICP among pregnant women in Hangzhou, China was 1.75%, this was lower than that in Chitwan Medical College in Nepal (2.50%) [21] but higher than in three tertiary hospitals in Australia (0.70%) [22], where the incidence rate was 0.60%/year [23].

The results of this study showed that pregnant women who were hepatitis B carriers were at a higher risk of ICP. Jiang et al. [24] reported that pregnant women infected by HBV have a higher risk of ICP and ICP patients are more susceptible to the risk of HBV infection. In another study, Xiong et al. [25] suggested that pregnant women receiving antiretroviral therapy, maternal HBV infection (HBsAg or HBsAg HBeAg) may increase the risk of ICP, but may not be associated with other pregnancy complications or neonatal outcomes. Similarly, Cai et al. [26] showed that chronic HBV infection during pregnancy may increase ICP (aOR = 1.700) and that pregnant women with an HBeAg-positive (aOR = 2.960) or HBeAg-negative (aOR = 1.520) status still have the risk of ICP. Our previous study also found that HBsAg-positive pregnant women in Hangzhou, China have a higher risk of ICP (aOR = 3.169) [19]. These studies demonstrated that pregnant women who were carriers of hepatitis B were more likely to develop ICP. Hence, the diagnosis and treatment of hepatitis B virus infection combined with ICP should be strengthened in clinical work to reduce the occurrence of related adverse pregnancy outcomes.

The results of this study further suggest that ICP may increase the incidence of shorter gestational days while reducing fetal macrosomia. A smaller number of gestational days results in earlier and lower-weight babies. In addition, preterm birth can also increase the risk of neonatal morbidity, some of which may require intensive care. However, studies by Friberg et al. [27] pointed out that the early induction of labor at 37 weeks of pregnancy seemed reasonable for high-risk ICP, without obvious maternal and fetal defects after induction, and can significantly reduce the mortality of ICP. Moreover, Shemer et al. [28] also found that women who experienced induced labor had a more than 50.00% lower risk of having an emergency cesarean delivery than those who did not. This may also confirm our finding that preterm premature rupture of membranes is a protective factor for ICP.

A short gestational age is also an important independent factor to predict adverse perinatal outcomes in patients with ICP. Madazli et al. [29] conducted a binary logistic regression analysis and showed that gestational age at diagnosis could predict preterm birth (OR = 2.300). Patients diagnosed before 30 weeks of gestation had significantly higher rates of respiratory distress syndrome (RDS), IUGR, fetal distress, and preterm birth than those diagnosed after 34 weeks of gestation (*P* < 0.01). With regards to determining the optimal gestational age for delivery in patients with ICP, Lo et al. [30] showed that immediate delivery at 36 weeks in women with ICP was the optimal delivery strategy. However, Alsulyman et al. [9] reported no differences between their two study groups in terms of mean gestational age at delivery (38.50 weeks vs. 38.80 weeks), birth weight (3216 g vs. 3277 g) and preterm birth rate (14.00% vs. 7.60%); these findings conflict with our present results. Few studies have been published that relate to the correlation between parity and ICP.

Table 1 shows that the ICP rate for advanced maternal age (9.91%) was slightly lower than that of younger pregnant women (10.26%), although this difference was not statistically significant (*P* > 0.05). The rate of cesarean section for ICP pregnant women with ICP (51.31%) was significantly higher than that of non-ICP women (31.60%) (*P* < 0.001). Table 6 shows that there was statistical difference in the rate of the cesarean section between the two groups according to multivariate binary logistic regression analysis (*P* < 0.001). In contrast, Heinonen et al. [31] reported different results, demonstrating that the risk of ICP increased in elderly pregnant women and that delivery by cesarean section (25.30%) was higher than in the general obstetric population (15.80%), which was similar to the results of our study. Studies have found that cesarean delivery is linked with an increased risk of pregnancy-related diseases. For example, cesarean section was associated with a sevenfold increase in the risk of HDP without overt proteinuria and a twofold increased risk of GH with overt proteinuria [32]. Reports have also pointed out that the odds of unexplained stillbirths with a history of cesarean section are significantly higher than those with a history of vaginal delivery [33]. Current studies on addressing the association between cesarean section and ICP are still limited. However, studies have shown that ICP is associated with an increased risk of PE [34]. ICP shares similar risk factors with PE during pregnancy, such as maternal age and multiple gestations [35–37]. Similarly, this study found that ICP may increase the incidence of cesarean section.

ICP is strongly associated with fetal distress and neonatal asphyxia [38]. Multiple animal models have shown that bile acids can cause severe chemical pneumonitis and pulmonary edema [8]. Zecca et al. [39] reported that elevated bile acid levels could affect alveolar enzyme function, thus leading to decreased surfactant levels and subsequent RDS. However, the incidence of fetal distress in this study was not significantly different (9.01% vs. 9.35%, *P* > 0.05). In addition, Table 3 shows that the incidence of hyperlipidemia in the ICP group was higher than that in the non-ICP group (5.96% vs. 3.80%) while Table 5 shows that hyperlipidemia (aOR = 1.602) is a risk factor for ICP. Zhang et al. and Martineau et al. [40, 41] both reported that ICP was associated with impaired glucose tolerance, dyslipidemia, and accelerated fetal growth. These authors also found that maternal blood lipid levels throughout pregnancy were significantly correlated with GDM, HDP, and ICP.

A large retrospective cohort study confirmed that mild or severe, stillbirth-free ICP was generally favorable for pregnancy outcomes, which may be possibly secondary to aggressive medical management. Moreover, there are a large proportion of pregnant women affected compared to the general population in terms of pregnancy outcomes such as GDM, PE, and/or spontaneous preterm birth [23]. Data from Wikstrom et al. [36] confirmed an increased risk of preterm birth, but not stillbirth, in actively managed cases of ICP. The high incidence of GDM and PE is a new finding that needs to be considered in the management of ICP pregnancies. Similarly, Martineau et al. [42] also showed that ICP is associated with an increased risk of GDM. Our findings suggested a correlation between ICP and gestational hypertension and gestational diabetes (*P* < 0.05).

In this study, we investigated the relationship between ICP and pregnancy outcomes in Hangzhou. Although we studied a relatively large sample size, there are some limitations that need to be considered. First, we excluded twin or multiple pregnancies and IVF infants from this study, this was because twin pregnancies and IVF infants have a higher incidence of ICP than singleton and non-IVF cases with the former group exhibiting, clinical symptoms and poorer perinatal outcomes [43]. Second, although we studied a larger sample size, these findings are only representative of the Hangzhou region of China. Third, it should be noted that very little optimization work has been carried out on bile acids and liver function, due to the lack of specific data on liver function and bile acids in the data we analyzed. Finally, this study was a retrospective study that included a large sample size of almost 40,000. Studies with larger sample sizes provide sufficient data for analysis and extrapolation to subsamples. However, it is worth pointing out that the small *P*-values in the results of this study may be an artifact of the large sample while leading to potentially questionable statistical significance, such as infant length, infant weight and maternal BMI, which are contrary to some research findings and our own perceptions, see Table 2 and Table 5. This is one of the limitations of this study. An information systems study noted that almost half of the recent large sample papers relied on low *p*-values and that most authors failed to recognize the potential impact of large samples on *P*-values. Regarding how to take advantage of these large samples without falling victim to falling *P*-values, Lin et al. [44] suggested that researchers modeling large samples should not simply rely on the direction of the regression coefficients and low *P*-values to support their hypotheses. The advantages and challenges of large sample studies are still being debated. We should focus more on the practical significance of the study results and properly evaluate the validity of statistical significance, which may be one of our solutions. Further effort is required to understand the impact of different concentrations of bile acid status on maternal outcomes. Furthermore, future studies should include longer follow-up periods, more variables, and larger sample sizes.

## Conclusions

In conclusion, the incidence of ICP in Hangzhou, China was 1.75%. Pregnant women who carried hepatitis B were at higher risk of ICP. Moreover, pregnant women with PE, thrombocytopenia, GH, hyperlipidemia, and GDM are at higher risk of ICP. ICP is also associated with adverse pregnancy outcomes and may increase the incidence of shorter gestational days and non-vaginal delivery methods such as cesarean section, while reducing the incidence of premature rupture of membranes and fetal macrosomia. Therefore, there is an obligation to closely follow and monitor pregnant women with ICP combined with these related risk factors and pregnancy outcomes. Further research and follow-up are required in the future. We suggest that women with ICP and any associated risk factors should be followed up according to local protocols.

## Supplementary Information

**Additional file 1**: Raw data of the 39,244 pregnant women participating in this study.

## Data Availability

All data generated or analyzed during this study are included in this published article and its Additional file 1.

## Abbreviations

ICP: Intrahepatic cholestasis of pregnancy
HBsAg+: Hepatitis B virus carriers
OR: Odds ratio
LBW: Low birth weight
IUGR: Intrauterine growth retardation
BMI: Body Mass Index
MAP: Maternal mean arterial pressure
HDP: Hypertensive disorders of pregnancy
GDM: Gestational diabetes mellitus
IVF: In vitro fertilization
RDS: Respiratory distress syndrome
